# Konjac Ceramide Induces Semaphorin 3A Expression via the MAPK/AP-1 Signaling Axis and RORα in Normal Human Epidermal Keratinocytes

**DOI:** 10.3390/biom16050755

**Published:** 2026-05-21

**Authors:** Mirei Fujita, Yayoi Kamata, Nanami Tanemoto, Nobuaki Takahashi, Mitsutoshi Tominaga, Kenji Takamori

**Affiliations:** 1 Juntendo Itch Research Center (JIRC), Institute for Environmental and Gender-Specific Medicine, Juntendo University Graduate School of Medicine, 2-1-1 Tomioka, Urayasu 279-0021, Chiba, Japan; 2 Department of Dermatology, Juntendo University Urayasu Hospital, 2-1-1 Tomioka, Urayasu 279-0021, Chiba, Japan

**Keywords:** konjac ceramide, semaphorin 3A, keratinocytes, gene expression, itch

## Abstract

Epidermal hyperinnervation is a major cause of intractable itch in barrier dysfunction conditions such as atopic dermatitis. Keratinocyte-derived semaphorin 3A (Sema3A) suppresses epidermal hyperinnervation, but its expression is markedly reduced in barrier-disrupted skin. Although konjac ceramide (kCer) has been reported to act as a Sema3A-like ligand, the mechanisms by which it regulates Sema3A expression in keratinocytes remain unclear. Normal human epidermal keratinocytes (NHEKs) were treated with kCer, konjac glucosylceramide (kGlcCer), or C24 ceramide. Sema3A mRNA and protein levels were assessed by quantitative real-time PCR and enzyme-linked immunosorbent assay, respectively. The involvement of intracellular signaling was examined using mitogen-activated protein kinase (MAPK) inhibitors, activator protein-1 (AP-1) inhibitors, retinoic acid-related orphan receptor alpha (RORα) inverse agonists, and siRNAs targeting c-Jun, c-Fos, and RORα. kCer induced Sema3A expression in NHEKs more potently than kGlcCer or C24 ceramide and promoted Sema3A protein secretion. Pharmacological inhibition or genetic knockdown of MEK1/2, JNK, AP-1 components, or RORα significantly attenuated kCer-induced *Sema3A* expression, indicating involvement of the MAPK/AP-1 signaling axis and RORα. kCer upregulates Sema3A expression in human keratinocytes through MAPK/AP-1 signaling and RORα, suggesting it may represent a promising antipruritic agent for epidermal hyperinnervation associated with skin barrier dysfunction.

## 1. Introduction

The skin epidermis functions as a permeability barrier that protects the body from environmental factors such as allergens, microbes, and chemicals. This barrier is maintained by keratinocyte differentiation and intercellular lipids, including ceramides, which are essential for water retention and protection against external insults [1,2,3]. Disruption of the epidermal barrier is a major predisposing factor for inflammatory skin diseases such as atopic dermatitis (AD).

Epidermal hyperinnervation is a characteristic feature of barrier-disrupted skin, including that observed in xerosis and AD, and contributes to the development of intractable itch [4]. This abnormal increase in intraepidermal nerve fibers is thought to result from an imbalance between nerve growth factors and nerve repulsion factors. Semaphorin 3A (Sema3A), a nerve repulsion factor, plays a critical role in suppressing epidermal hyperinnervation [5]. Previous studies have demonstrated that Sema3A is primarily produced by keratinocytes, that its expression is decreased in barrier-disrupted skin, and that supplementation of Sema3A ameliorates itch-related symptoms [4,6]. However, despite its therapeutic potential, the molecular mechanisms regulating Sema3A expression in epidermal keratinocytes remain poorly understood.

**Figure 1:**
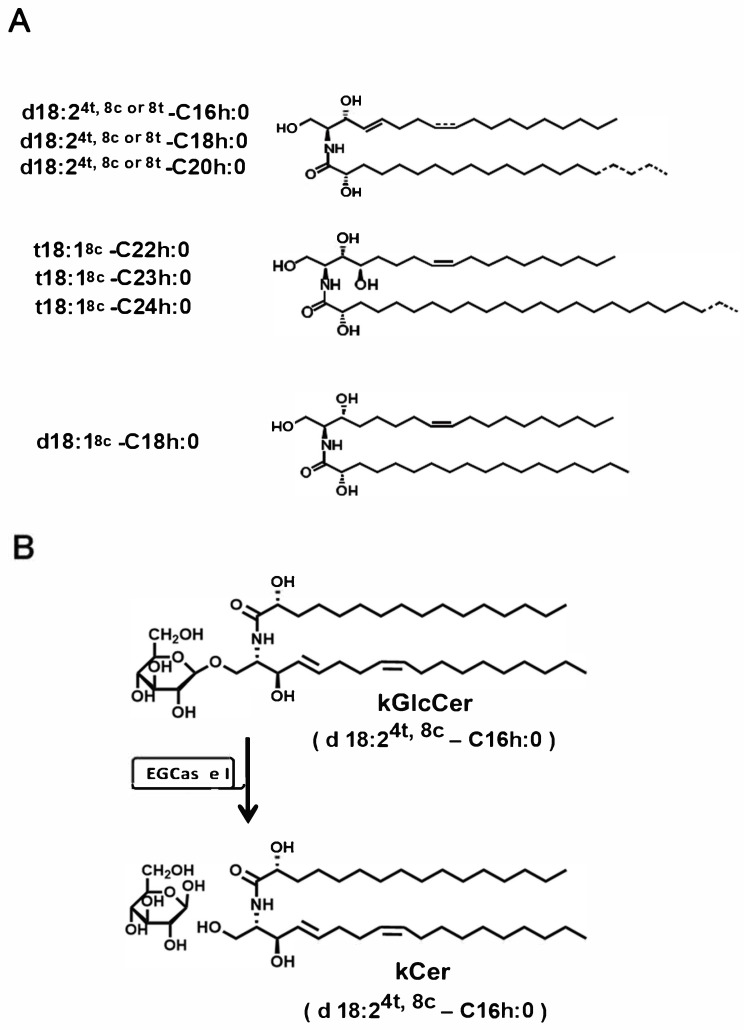
Chemical structure of kCer and EGCase I-catalyzed deglucosylation for kCer preparation. (**A**) Molecular species of kCer generated by EGCase treatment of kGlcCer. No significant differences in molecular species composition were observed between kCer and kGlcCer, reflecting the substrate specificity of EGCase. (**B**) Schematic of the EGCase-mediated reaction of kGlcCer. Plant-type ceramides can be prepared by treating plant-derived GlcCer with EGCase I.

Ceramides have been studied as supplements for the prevention of lifestyle-related diseases [7]. In mammalian cells, ceramides consist of sphingosine and fatty acids and are present as free ceramides in cell membranes [2,3]. In contrast, in plant tissues, ceramides are mainly present as glucosylceramides (GlcCer) and glycosylinositol phosphoceramides [8]. Structural differences between animal- and plant-derived ceramides are attributed to the diversity of long-chain bases [9]. Molecular species of konjac ceramide (kCer) are characterized by combinations of long-chain bases (d18:2, t18:1) and 2-hydroxyl fatty acids (C16:0, C18:0, C20:0, C22:0, C23:0, and C24:0) (Figure 1A).

Konjac, prepared from the tuber of *Amorphophallus konjac*, is a Japanese traditional food rich in glucomannan and GlcCer. Onishi et al. reported that oral administration of konjac glucomannan suppressed scratching behavior and skin inflammation in NC/Nga mice [10]. Furthermore, oral intake of *A. konjac*-derived glucosyl ceramide (kGlcCer) improved barrier function and reduced allergic skin reactions in patients with atopic eczema [11,12]. kCer is chemoenzymatically produced by deglucosylation of kGlcCer using endoglycoceramidase I (EGCase I) (Figure 1B) [13,14]. Previous studies have shown that kCer binds to the Sema3A receptor neuropilin-1 (Nrp1) and inhibits nerve growth factor-induced neurite outgrowth via Sema3A-like activity [13,15,16,17]. However, it remains unclear whether kCer directly regulates endogenous *Sema3A* gene expression in keratinocytes independently of its ligand-like activity, and what intracellular signaling mechanisms are involved in this process. In particular, it is unknown whether kCer activates specific transcriptional pathways leading to Sema3A upregulation. Therefore, in this study, we aimed to determine whether kCer directly induces endogenous Sema3A expression in normal human epidermal keratinocytes (NHEKs) and to elucidate the underlying intracellular signaling mechanisms. By clarifying the molecular basis of kCer-induced Sema3A expression, we sought to provide new insights into its potential as an antipruritic agent targeting epidermal hyperinnervation.

## 2. Materials and Methods

### 2.1. Materials and Reagents

kCer was kindly provided by Dr. Seigo Usuki (Hokkaido University) [13]. C24Cer was purchased from Avanti Polar Lipids (Alabaster, AL, USA). kGlcCer was from Nagara Science (NS170303, Gifu, Japan). T-5224 was purchased from AdooQ Bioscience (Irvine, CA, USA). SR3335 (a retinoic acid -related orphan receptor alpha [RORα] inverse agonist) was obtained from Cayman Chemical (Ann Arbor, MI, USA). Antibodies against p44/42 MAPK (extracellular signal-regulated kinase [ERK]1/2), stress-activated protein kinase/c-Jun N-terminal kinase (SAPK/JNK), and p38 MAPK, as well as phospho-specific antibodies against p44/42 MAPK (ERK1/2; Thr202/Tyr204), SAPK/JNK (Thr183/Tyr185), and p38 MAPK (Thr180/Tyr182), were purchased from Cell Signaling Technology (Beverly, MA, USA). PD98059 (a MAPK/ERK kinase [MEK]1/2 inhibitor) and SP600125 (a JNK inhibitor) were also obtained from the same supplier. The Cell Counting Kit-8 (CCK-8) was purchased from Dojindo (Kumamoto, Japan). WIDE-VIEW Prestained Protein Size Marker III was obtained from Fujifilm (Tokyo, Japan). BMS-582949 (a p38 inhibitor) was purchased from Selleck Chemicals (Houston, TX, USA). Ex Taq DNA polymerase, the PrimeScript RT reagent kit, and TB Green Premix Ex Taq were obtained from Takara (Shiga, Japan). All other chemicals were of analytical grade.

### 2.2. Cell Culture

NHEKs derived from an adult epidermis were purchased from Lonza (Basel, Switzerland). NHEKs were cultured in keratinocyte basal medium-Gold containing 0.1 mM calcium, supplemented with keratinocyte growth medium-Gold SingleQuots (Lonza, Basel, Switzerland). Cells were incubated at 37 °C in a humidified atmosphere containing 5% CO_2_ and used within three passages. Ceramides were dissolved in dimethyl sulfoxide (DMSO) to prepare stock solutions, with gentle warming and sonication. To examine the effects of ceramides on Sema3A expression, subconfluent NHEKs were treated with 25–100 μM C24Cer, kGlcCer, or kCer in the presence of 0.1 mM calcium. After 48 h, cells were harvested for total RNA isolation or culture supernatants were collected. To evaluate the effects of inhibitors, subconfluent NHEKs were pretreated with each inhibitor or inverse agonist for 1 h, followed by co-treatment with kCer and the respective inhibitor for 24 h. Cells were then harvested for RNA isolation or protein analysis.

The murine keratinocyte cell line PAM212 was provided by Dr. Toshihiko Hibino (Shiseido Co., Ltd., Tokyo, Japan). PAM212 cells were cultured in Dulbecco’s Modified Eagle’s Medium (low-glucose; Sigma Aldrich, Munich, Germany) supplemented with 10% fetal bovine serum and penicillin (100 IU/mL)-streptomycin (100 μg/mL) (Sigma-Aldrich, St. Louis, MO, USA). Cells were incubated at 37 °C in 5% CO_2_ prior to total RNA isolation. The transcription level of Sema3A was analyzed by quantitative real-time PCR. Information on the mouse primers used is shown in Table S2.

### 2.3. siRNA Transfection

NHEKs were transfected with 40 nM siGENOME SMARTpool siRNAs targeting c-Jun, c-Fos, and/or RORα (Horizon Discovery, Cambridge, UK) using Lipofectamine RNAiMAX (Thermo Fisher Scientific, Waltham, MA, USA), according to the manufacturer’s instructions. After 48 h, cells were treated with 25 µM kCer for an additional 24 h prior to RNA isolation. A non-targeting siRNA was used as a control.

### 2.4. Quantitative Real-Time PCR Analysis

Gene expression levels of Sema3A and related factors were analyzed by quantitative real-time PCR. Total RNA was extracted using an RNeasy Mini Kit (Qiagen, Hilden, Germany). cDNA synthesis and PCR amplification were performed using the PrimeScript RT reagent kit and TB Green Premix Ex Taq (Takara, Shiga, Japan) on a QuantStudio 5 system (Thermo Fisher Scientific, Waltham, MA, USA). Primer sequences are listed in Table S1. Amplification specificity was confirmed by melting curve analysis. mRNA expression levels were normalized to ribosomal protein S18 (*RPS18*) and are presented relative to the untreated control.

### 2.5. Enzyme-Linked Immunosorbent Assay (ELISA)

After 48 h treatment with 25–100 μM C24Cer, kGlcCer, or kCer, culture supernatants were collected, and Sema3A protein levels were measured using a human Sema3A ELISA kit (USCN Life Sciences, Wuhan, China), according to the manufacturer’s instructions.

### 2.6. Cell Viability Assay

Cell viability was assessed using the Cell Counting Kit-8 (CCK-8; Dojindo). NHEKs were seeded at a density of 1 × 10^4^ cells per well in 96-well plates. After 24 h, cells were treated with 25–100 μM C24Cer, kGlcCer, or kCer. Following 48 h incubation, CCK-8 solution was added and incubated at 37 °C for 3 h. Absorbance at 450 nm was measured using an ARVO X4 multilabel plate reader (PerkinElmer, Waltham, MA, USA).

### 2.7. Western Blot Analysis

Cell lysates were prepared using M-PER mammalian protein extraction reagent supplemented with protease and phosphatase inhibitor cocktails (Thermo Fisher Scientific, Waltham, MA, USA). Equal amounts of protein (5 µg) were separated on 10% e-PAGEL gels (ATTO, Tokyo, Japan) and electrophoresed at 20 mA per gel. Proteins were then transferred onto Immobilon-P PVDF membranes (Millipore, Burlington, MA, USA) using a Powered Blot system (ATTO, Tokyo, Japan). Membranes were blocked with 2% bovine serum albumin for 1 h at room temperature and incubated with primary antibodies at 4 °C overnight. After washing with Tris-buffered saline containing Tween 20 (TBST), membranes were incubated with horseradish peroxidase-conjugated goat anti-rabbit IgG (H+L) secondary antibody (Thermo Fisher Scientific) for 1 h at room temperature. Signals were detected using SuperSignal West Pico PLUS chemiluminescent substrate and visualized with an Amersham Imager 600 (GE Healthcare, Chicago, IL, USA). Anti-β-actin (ProteinTech, Rosemont, IL, USA) was used as a loading control. Semi-quantification of the bands was performed using Image J 1.54g.

### 2.8. Statistical Analysis

Statistical analyses were performed using Student’s *t*-test or one-way analysis of variance followed by Dunnett’s or Tukey’s multiple comparison tests, as appropriate. Analyses were conducted using GraphPad Prism 9 (GraphPad Software, La Jolla, CA, USA).

### 2.9. Generative AI Tools

An artificial intelligence tool (Microsoft Copilot) was used to assist with language editing to improve the clarity of the manuscript. All scientific content, data interpretation, and conclusions were generated and verified by the authors.

## 3. Results

### 3.1. Induction of Sema3A Expression by kCer

**Figure 2:**
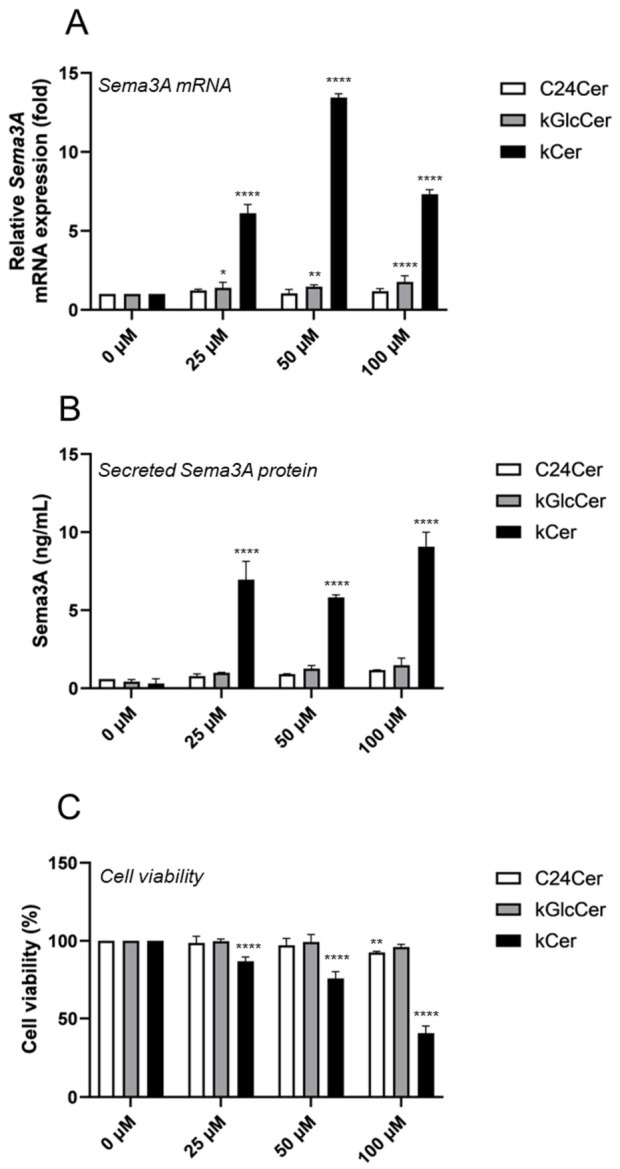
Effects of various ceramides on Sema3A expression in normal human epidermal keratinocytes (NHEKs). NHEKs were incubated with various ceramides (C24Cer, kGlcCer, or kCer) at 25, 50, or 100 μM at 37 °C for 48 h. (**A**) *Sema3A* mRNA expression levels were analyzed by quantitative real-time PCR and normalized to RPS18. (**B**) Secreted Sema3A protein levels in culture supernatants were measured by ELISA. (**C**) Cell viability was assessed using Cell Counting Kit-8. All results are expressed as the mean ± SD of three independent experiments. * *p* < 0.05, ** *p* < 0.01, **** *p* < 0.0001 (vs. 0 μM; one-way ANOVA followed by Dunnett’s test).

To first evaluate the biological effect of kCer, we examined its impact on Sema3A expression in cultured keratinocytes. Among the tested ceramides (C24Cer, kGlcCer, and kCer), kCer was the most potent inducer of *Sema3A* mRNA expression in NHEKs (Figure 2A) and significantly increased Sema3A protein secretion in a dose-dependent manner (Figure 2B). In contrast, kGlcCer also increased *Sema3A* mRNA levels, but to a lesser extent than kCer, whereas C24Cer had no significant effect (Figure 2A,B). Notably, kCer did not induce *Sema3A* mRNA expression in the murine keratinocyte cell line PAM212 (Figure S1), suggesting a species-specific response.

Cell viability analysis revealed that kCer reduced NHEK viability in a dose-dependent manner after 48 h (Figure 2C), whereas C24Cer and kGlcCer showed minimal cytotoxic effects. Importantly, robust induction of *Sema3A* expression was observed at 25 μM kCer, a concentration at which cell viability was largely preserved, suggesting that Sema3A upregulation is not merely a consequence of nonspecific cytotoxic stress.

### 3.2. Signaling Pathways Involved in kCer-Induced Sema3A Expression

**Figure 3:**
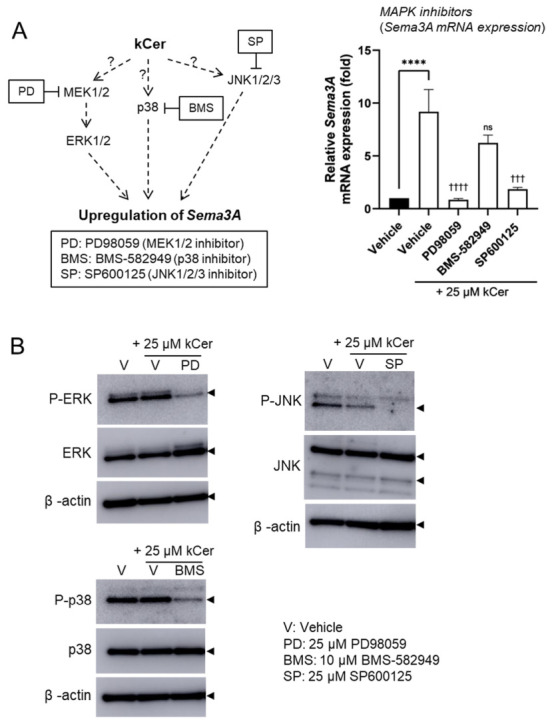
Involvement of the MAPK signaling axis in kCer-induced upregulation of Sema3A expression in NHEKs. (**A**) The panel on the left shows the site of action of the inhibitor. Question marks indicate that the pathway is not yet well understood. The right panel shows *Sema3A* mRNA expression following kCer treatment in the presence of a MAPK inhibitor. Effects of MAPK inhibitors on kCer-induced *Sema3A* expression were analyzed by quantitative real-time PCR. NHEKs were treated with 25 μM kCer in the presence or absence of MAPK inhibitors (25 μM PD98059, 10 μM BMS-582949, or 25 μM SP600125) for 24 h. Total RNA was then isolated and Sema3A expression was quantified. Values are expressed relative to the vehicle control (without kCer = 1). (**B**) Western blot analysis of ERK1/2, p38, and JNK1/2/3 phosphorylation in NHEKs following stimulation with 25 μM kCer and inhibition with the indicated MAPK inhibitors. All results are expressed as the mean ± SD of three independent experiments. **** *p* < 0.0001 (vs. vehicle without kCer; one-way ANOVA with Dunnett’s test). ^†††^ *p* < 0.001, ^††††^ *p* < 0.0001, ns (not significant) (vs. vehicle with 25 μM kCer; one-way ANOVA with Tukey’s test). Western blot original images can be found in Supplementary Materials.

**Figure 4:**
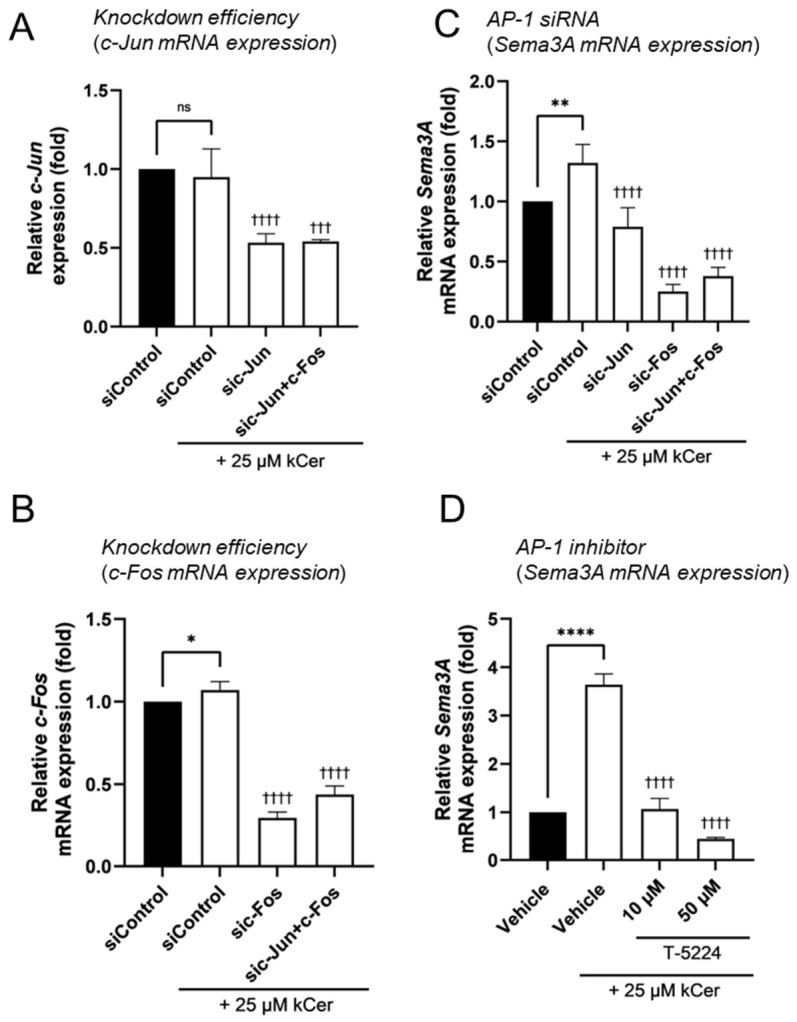
Involvement of the transcription factor AP-1 in kCer-induced upregulation of Sema3A expression in NHEKs. NHEKs were transfected with siRNA (40 nM), cultured for 48 h, and then treated with 25 μM kCer for 24 h prior to RNA isolation. (**A**,**B**) Knockdown efficiencies of *c-Jun* (**A**) and *c-Fos* (**B**). (**C**) *Sema3A* mRNA expression levels were analyzed by real-time PCR. Values are expressed relative to siControl without kCer (=1). (**D**) Effects of the AP-1 inhibitor T-5224 (10 or 50 μM) on kCer-induced *Sema3A* expression were evaluated by real-time PCR. Values are expressed relative to the vehicle control (=1). All results are expressed as the mean ± SD of three independent experiments. * *p* < 0.05, ** *p* < 0.01, **** *p* < 0.0001, ns (not significant) (vs. siControl without kCer); ^†††^ *p* < 0.001, ^††††^ *p* < 0.0001 (vs. siControl with 25 μM kCer); one-way ANOVA followed by Tukey’s test.

We previously reported that the MEK1/2–ERK1/2 pathway is involved in *Sema3A* expression in NHEKs [18]. To elucidate the molecular mechanisms underlying kCer-induced Sema3A upregulation, we next investigated the involvement of MAPK signaling pathways in NHEKs. Treatment with 25 μM kCer significantly induced *Sema3A* expression; however, this induction was markedly attenuated by the MEK1/2 inhibitor PD98059 and the JNK inhibitor SP600125 (Figure 3A). The p38 inhibitor BMS-582949 also suppressed kCer-induced *Sema3A* expression, although its effect was less pronounced. Consistent with these findings, ERK1/2, p38, and JNK were phosphorylated under basal conditions, and their phosphorylation levels were reduced in the presence of the respective inhibitors (Figure 3B, Figure S2). Given the established role of AP-1 in *Sema3A* transcription [18,19], we next examined the involvement of AP-1 components. siRNA-mediated knockdown of c-Jun and/or c-Fos significantly suppressed kCer-induced *Sema3A* expression (Figure 4C), and the AP-1 inhibitor T-5224 also inhibited this induction in a dose-dependent manner (Figure 4D).

### 3.3. Role of RORα in kCer-Induced Sema3A Expression

**Figure 5:**
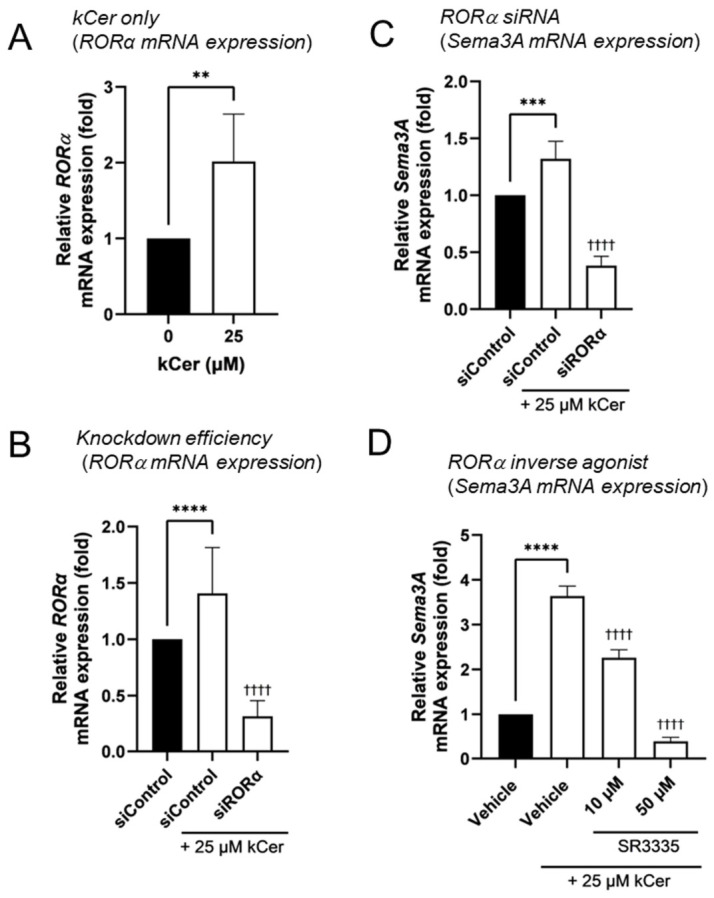
Involvement of RORα in kCer-induced upregulation of Sema3A expression in NHEKs. (**A**) NHEKs were incubated with 25 μM kCer at 37 °C for 24 h. Total RNA was isolated, and *RORα* mRNA expression was analyzed by real-time PCR. (**B**,**C**) NHEKs were transfected with siRNA (40 nM), cultured for 48 h, and then treated with 25 μM kCer for 24 h. Knockdown efficiency of *RORα* (**B**) and *Sema3A* mRNA expression (**C**) were evaluated by real-time PCR. (**D**) Effects of the RORα-selective inverse agonist SR3335 (10 or 50 μM) on kCer-induced *Sema3A* expression were analyzed by real-time PCR. All results are expressed as the mean ± SD of three independent experiments. ** *p* < 0.01 (vs. 0 μM kCer); *** *p* < 0.001, **** *p* < 0.0001 (vs. siControl without kCer); ^††††^ *p* < 0.0001 (vs. siControl with 25 μM kCer); one-way ANOVA followed by Dunnett’s or Tukey’s test as appropriate.

Given the previously reported role of RORα in regulating Sema3A expression [20], we further investigated its involvement in kCer-induced *Sema3A* upregulation. kCer treatment significantly increased *RORα* mRNA expression in NHEKs (Figure 5A). siRNA-mediated knockdown of RORα markedly reduced kCer-induced *Sema3A* expression (Figure 5C). Similarly, treatment with the RORα inverse agonist SR3335 significantly attenuated this effect in a dose-dependent manner (Figure 5D). These results indicate that RORα plays at least a partial role, in the transcriptional regulation of *Sema3A* induced by kCer.

## 4. Discussion

The present study demonstrates that kCer acts as an upregulator of Sema3A expression in NHEKs. Our findings show that kCer-induced Sema3A expression is mediated through the MAPK/AP-1 signaling axis and is partially regulated by RORα. These results provide new insight into the molecular mechanisms underlying the potential antipruritic effects of kCer. MAPK signaling pathways play essential roles in keratinocyte function, including proliferation, differentiation, and stress responses [21,22,23]. In this study, pharmacological inhibition of MEK1/2 and JNK markedly attenuated kCer-induced *Sema3A* expression, and AP-1 inhibition or knockdown of its components (c-Jun and c-Fos) significantly reduced this effect (Figure 3A and Figure 4C,D). These findings suggest that kCer enhances *Sema3A* transcription at least in part via the MAPK/AP-1 axis. Notably, kCer did not strongly increase MAPK phosphorylation beyond basal levels, indicating that constitutive MAPK activity may play a permissive role in this process rather than acting as a primary activating signal. This mode of action differs from the previously reported ligand-like activity of kCer via neuropilin-1 and highlights a novel intracellular mechanism.

**Figure 6:**
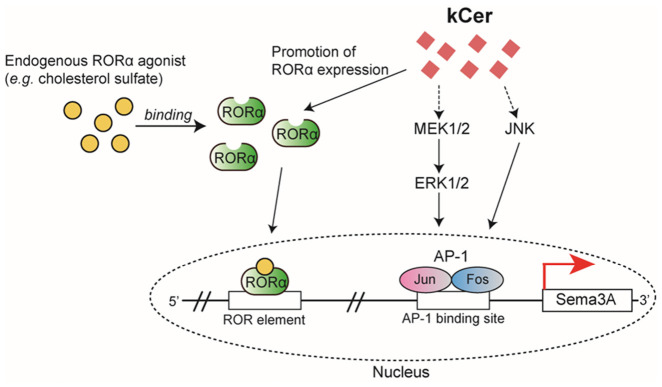
Proposed mechanism for kCer-induced Sema3A expression in NHEKs. kCer induces Sema3A mRNA expression via activation of ERK1/2 and JNK, leading to AP-1 binding to the proximal promoter of the Sema3A gene. In parallel, kCer enhances RORα expression, promoting its binding to the ROR response element in the promoter region. In the presence of an RORα agonist, kCer further enhances Sema3A expression in NHEKs. Solid lines indicate interactions whose mechanisms are established. Dashed lines indicate that the direct interaction is uncertain.

In addition to MAPK/AP-1 signaling, our results demonstrate that RORα contributes to kCer-induced Sema3A expression. RORα is a member of the nuclear receptor superfamily that binds to specific DNA sequences known as ROR response elements [24,25]. kCer increased *RORα* mRNA expression, and both siRNA-mediated knockdown and pharmacological inhibition of RORα significantly attenuated Sema3A induction (Figure 5C,D). These findings are consistent with previous reports indicating that RORα regulates *Sema3A* transcription [20]. Taken together, our data suggest that kCer promotes Sema3A expression through MAPK/AP-1 signaling and RORα-dependent transcriptional regulation (Figure 6).

An important aspect of the present study is the relationship between Sema3A induction and cytotoxicity. kCer reduced NHEK viability in a dose-dependent manner; however, robust Sema3A induction was observed at 25 μM, a concentration at which cell viability was largely preserved. This suggests a potential in vitro therapeutic window in which kCer can effectively induce Sema3A expression without causing substantial cytotoxic effects. At higher concentrations, increased cytotoxicity and putative negative feedback mechanisms may contribute to the observed non-linear dose–response relationship.

Interestingly, kCer did not induce *Sema3A* expression in the murine keratinocyte cell line PAM212, indicating a species-specific response (Figure S1). This may reflect variations in transcriptional regulation between species, such as differences in RORα expression levels, cofactor interactions, or conservation of regulatory elements within the *Sema3A* promoter. Further studies will be required to clarify the molecular basis of this species specificity.

Among the ceramides examined in this study, kCer exhibited the most potent effect on Sema3A expression, whereas kGlcCer showed a weaker effect and C24Cer had minimal activity (Figure 2A,B). These findings suggest that Sema3A induction is not a general property of ceramides but may depend on specific structural features unique to kCer, such as its long-chain base composition or hydroxylated fatty acid moieties. This structural specificity may underlie the potential advantage of kCer as an antipruritic agent.

The oral intake of plant-derived ceramides, such as those from maize, beet, and konjac, has been shown to improve epidermal barrier function in both animal models and humans [26]. Oral administration of kGlcCer improves barrier function and alleviates symptoms such as dryness and itching in healthy individuals and AD patients [11,12,27]. Similarly, soybean GlcCer suppressed inflammation and itch-related scratching in a mouse model of contact dermatitis [28]. Limited information is available regarding the topical application of plant ceramides. Previous studies have shown that topical maize GlcCer suppresses UVA-induced photoaging in hairless mice [29], and that ceramide-containing ointments prevent the downregulation of epidermal differentiation markers in barrier-disrupted skin models, although they do not directly alter gene expression [30]. Because ceramides are components of stratum corneum intercellular lipids, topically applied ceramides may not effectively penetrate into viable epidermal layers (from stratum basale to stratum granulosum). Given that kCer has a molecular weight of approximately 700 Da, it is unlikely to penetrate intact skin (>500 Da rule) [31]. Therefore, oral administration of kCer may be more effective than topical application. *Sema3A* mRNA induction at 100 µM kCer was weaker than at 50 µM (Figure 2A). Our preliminary data indicate that high concentrations of recombinant Sema3A suppress endogenous *Sema3A* mRNA expression (Figure S3A) and inhibit calcium-induced *Sema3A* upregulation (Figure S3B). These findings suggest that excessive Sema3A production may trigger a negative feedback mechanism that suppresses its own expression.

This study has several limitations. First, all experiments were conducted using NHEKs, which may not fully recapitulate the complexity of in vivo skin environments. Second, no in vivo validation was performed to confirm the physiological relevance of kCer-induced Sema3A expression. Third, species-specific differences were observed, and the underlying mechanisms remain unclear. Finally, although Sema3A induction was observed under conditions with minimal cytotoxicity, the potential influence of higher-dose cytotoxic effects cannot be completely excluded. Future studies addressing these limitations will be important to further clarify the therapeutic potential of kCer.

## 5. Conclusions

In conclusion, kCer induces Sema3A expression through MAPK/AP-1 signaling and RORα in NHEKs. These findings suggest kCer is a promising candidate as an antipruritic agent for treating epidermal hyperinnervation associated with skin barrier dysfunction, including xerosis and AD.

## Data Availability

Data supporting the findings of this study are presented in the article and Supplementary Materials. Additional information is available upon reasonable request: e-mail: ktakamor@juntendo.ac.jp.

## Supplementary Materials

The following supporting information can be downloaded at: https://www.mdpi.com/article/10.3390/biom16050755/s1, Figure S1: Effects of kCer on *Sema3A* mRNA expression in the murine keratinocyte cell line PAM212; Figure S2: Western blot images used to generate Figure 3; Figure S3: Recombinant Sema3A protein suppresses *Sema3A* mRNA expression in NHEKs; Table S1: Human real-time PCR primers; Table S2: Murine real-time PCR primers.

## Abbreviations

AP-1	activator protein-1
AD	atopic dermatitis
kCer	konjac ceramide
EGCase	endoglycoceramidase
ERK	extracellular signal-regulated kinase
kGlcCer	konjac glucosylceramide
MAPK	mitogen-activated protein kinase
MEK	MAPK-ERK kinase
NHEKs	normal human epidermal keratinocytes
Sema3A	semaphorin 3A

## References

[1] Kabashima K. (2013). New concept of the pathogenesis of atopic dermatitis: Interplay among the barrier, allergy, and pruritus as a trinity. J. Dermatol. Sci..

[2] Sakai T., Hatano Y. (2025). Stratum corneum pH and ceramides: Key regulators and biomarkers of skin barrier function in atopic dermatitis. J. Dermatol. Sci..

[3] Feingold K.R. (2007). Thematic review series: Skin lipids. The role of epidermal lipids in cutaneous permeability barrier homeostasis. J. Lipid Res..

[4] Luo Y., Raible D., Raper J.A. (1993). Collapsin: A protein in brain that induces the collapse and paralysis of neuronal growth cones. Cell.

[5] Tominaga M., Takamori K. (2014). Itch and nerve fibers with special reference to atopic dermatitis: Therapeutic implications. J. Dermatol..

[6] Negi O., Tominaga M., Tengara S., Kamo A., Taneda K., Suga Y., Ogawa H., Takamori K. (2012). Topically applied semaphorin 3A ointment inhibits scratching behavior and improves skin inflammation in NC/Nga mice with atopic dermatitis. J. Dermatol. Sci..

[7] Baldwin H., Del Rosso J. (2024). Going Beyond Ceramides in Moisturizers: The Role of Natural Moisturizing Factors. J. Drugs Dermatol..

[8] Gronnier J., Germain V., Gouguet P., Cacas J.L., Mongrand S. (2016). GIPC: Glycosyl Inositol Phospho Ceramides, the major sphingolipids on earth. Plant Signal. Behav..

[9] Minamioka H., Imai H. (2009). Sphingoid long-chain base composition of glucosylceramides in Fabaceae: A phylogenetic interpretation of Fabeae. J. Plant Res..

[10] Onishi N., Kawamoto S., Ueda K., Yamanaka Y., Katayama A., Suzuki H., Aki T., Hashimoto K., Hide M., Ono K. (2007). Dietary pulverized konjac glucomannan prevents the development of allergic rhinitis-like symptoms and IgE response in mice. Biosci. Biotechnol. Biochem..

[11] Kimata H. (2006). Improvement of atopic dermatitis and reduction of skin allergic responses by oral intake of konjac ceramide. Pediatr. Dermatol..

[12] Miyanishi K., Shiono N., Shirai H., Dombo M., Kimata H. (2005). Reduction of transepidermal water loss by oral intake of glucosylceramides in patients with atopic eczema. Allergy.

[13] Usuki S., Tamura N., Sakai S., Tamura T., Mukai K., Igarashi Y. (2016). Chemoenzymatically prepared konjac ceramide inhibits NGF-induced neurite outgrowth by a semaphorin 3A-like action. Biochem. Biophys. Rep..

[14] Ishibashi Y., Kobayashi U., Hijikata A., Sakaguchi K., Goda H.M., Tamura T., Okino N., Ito M. (2012). Preparation and characterization of EGCase I, applicable to the comprehensive analysis of GSLs, using a rhodococcal expression system. J. Lipid Res..

[15] Usuki S., Tamura N., Yuyama K., Tamura T., Mukai K., Igarashi Y. (2018). Konjac Ceramide (kCer) Regulates NGF-Induced Neurite Outgrowth via the Sema3A Signaling Pathway. J. Oleo Sci..

[16] Usuki S., Tamura N., Tamura T., Mukai K., Igarashi Y. (2018). Characterization of Konjac Ceramide (kCer) Binding to Sema3A Receptor Nrp1. J. Oleo Sci..

[17] Usuki S., Tamura N., Tamura T., Higashiyama S., Tanji K., Mitsutake S., Inoue A., Aoki J., Mukai K., Igarashi Y. (2019). Konjac ceramide (kCer) regulates keratinocyte migration by Sema3A-like repulsion mechanism. Biochem. Biophys. Rep..

[18] Kamata Y., Tominaga M., Umehara Y., Honda K., Kamo A., Moniaga C.S., Komiya E., Toyama S., Suga Y., Ogawa H. (2020). Calcium-Inducible MAPK/AP-1 Signaling Drives Semaphorin 3A Expression in Normal Human Epidermal Keratinocytes. J. Investig. Dermatol..

[19] Fujita M., Kamata Y., Tanemoto N., Morita M., Tobita T., Zhao Q., Tominaga M., Takamori K. (2026). Parbendazole induces semaphorin 3A expression via JNK/c-Jun signaling pathway in normal human epidermal keratinocytes. J. Dermatol. Sci..

[20] Kamata Y., Tominaga M., Sakaguchi A., Umehara Y., Negi O., Ogawa H., Takamori K. (2015). Retinoid-related orphan receptor alpha is involved in induction of semaphorin 3A expression in normal human epidermal keratinocytes. J. Dermatol. Sci..

[21] Eckert R.L., Efimova T., Dashti S.R., Balasubramanian S., Deucher A., Crish J.F., Sturniolo M., Bone F. (2002). Keratinocyte survival, differentiation, and death: Many roads lead to mitogen-activated protein kinase. J. Investig. Dermatol. Symp. Proc..

[22] Hammouda M.B., Ford A.E., Liu Y., Zhang J.Y. (2020). The JNK Signaling Pathway in Inflammatory Skin Disorders and Cancer. Cells.

[23] Imajo M., Tsuchiya Y., Nishida E. (2006). Regulatory mechanisms and functions of MAP kinase signaling pathways. IUBMB Life.

[24] Solt L.A., Burris T.P. (2012). Action of RORs and their ligands in (patho)physiology. Trends Endocrinol. Metab..

[25] Solt L.A., Griffin P.R., Burris T.P. (2010). Ligand regulation of retinoic acid receptor-related orphan receptors: Implications for development of novel therapeutics. Curr. Opin. Lipidol..

[26] Tessema E.N., Gebre-Mariam T., Neubert R.H.H., Wohlrab J. (2017). Potential Applications of Phyto-Derived Ceramides in Improving Epidermal Barrier Function. Skin Pharmacol. Physiol..

[27] Heggar Venkataramana S., Puttaswamy N., Kodimule S. (2020). Potential benefits of oral administration of *Amorphophallus Konjac* glycosylceramides on skin health-a randomized clinical study. BMC Complement. Med. Ther..

[28] Yeom M., Kim S.H., Lee B., Han J.J., Chung G.H., Choi H.D., Lee H., Hahm D.-H. (2012). Oral administration of glucosylceramide ameliorates inflammatory dry-skin condition in chronic oxazolone-induced irritant contact dermatitis in the mouse ear. J. Dermatol. Sci..

[29] Shimada E., Aida K., Sugawara T., Hirata T. (2011). Inhibitory effect of topical maize glucosylceramide on skin photoaging in UVA-irradiated hairless mice. J. Oleo Sci..

[30] Huth S., Schmitt L., Marquardt Y., Heise R., Luscher B., Amann P.M., Baron J.M. (2018). Effects of a ceramide-containing water-in-oil ointment on skin barrier function and allergen penetration in an IL-31 treated 3D model of the disrupted skin barrier. Exp. Dermatol..

[31] Bos J.D., Meinardi M.M. (2000). The 500 Dalton rule for the skin penetration of chemical compounds and drugs. Exp. Dermatol..

