# Supplementary material for: Konjac Ceramide Induces Semaphorin 3A Expression via the MAPK/AP-1 Signaling Axis and RORα in Normal Human Epidermal Keratinocytes
Source: Biomolecules. 2026 May 21;16(5):755. doi: 10.3390/biom16050755 (PMC13205023; doi:10.3390/biom16050755)
Supplement: Supplementary file 1 [file biomolecules-16-00755-s001.zip › biomolecules-4288186-supplementary.pdf]

Supplementary materials and methods

Cell culture

The murine keratinocyte cell line PAM212 was provided by Dr. Toshihiko Hibino (Shiseido Co., Ltd.). PAM212 cells were cultured in Dulbecco's Modified Eagle's Medium (low-glucose; Sigma Aldrich, Munich, Germany) supplemented with 10% fetal bovine serum and penicillin (100 IU/mL)-streptomycin (100 g/mL) (Sigma-Aldrich). Cells were incubated at 37°C in 5% CO_2 prior to total RNA isolation. The transcription level of Sema3A was analyzed by quantitative real-time PCR. Information on the mouse primers used is shown in Table S2.

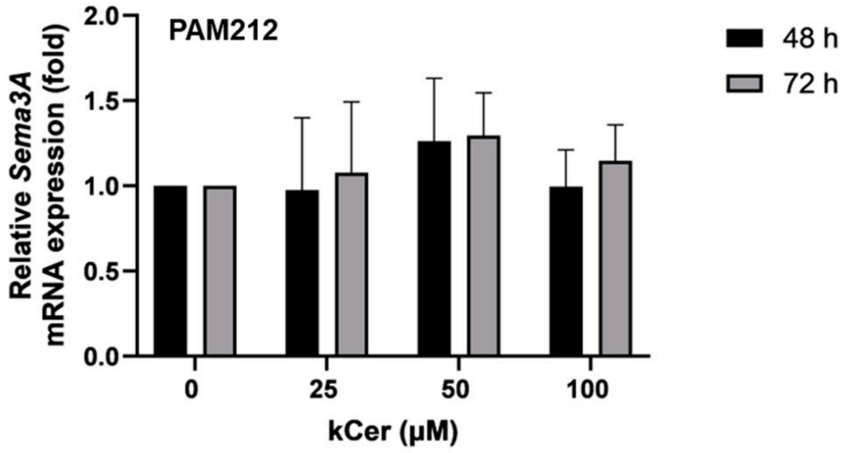

Figure S1. Effects of kCer on *Sema3A* mRNA expression in the murine keratinocyte cell line PAM212. PAM212 cells were incubated with kCer (25, 50, or 100 μM) at 37°C for 48 or 72 h. Sema3A mRNA expression was analyzed by quantitative real-time PCR. Expression levels were normalized to RPS18. All results are expressed as the mean ± S.D. of three independent experiments. (vs. 0 μM; one-way ANOVA followed by Dunnett's test).

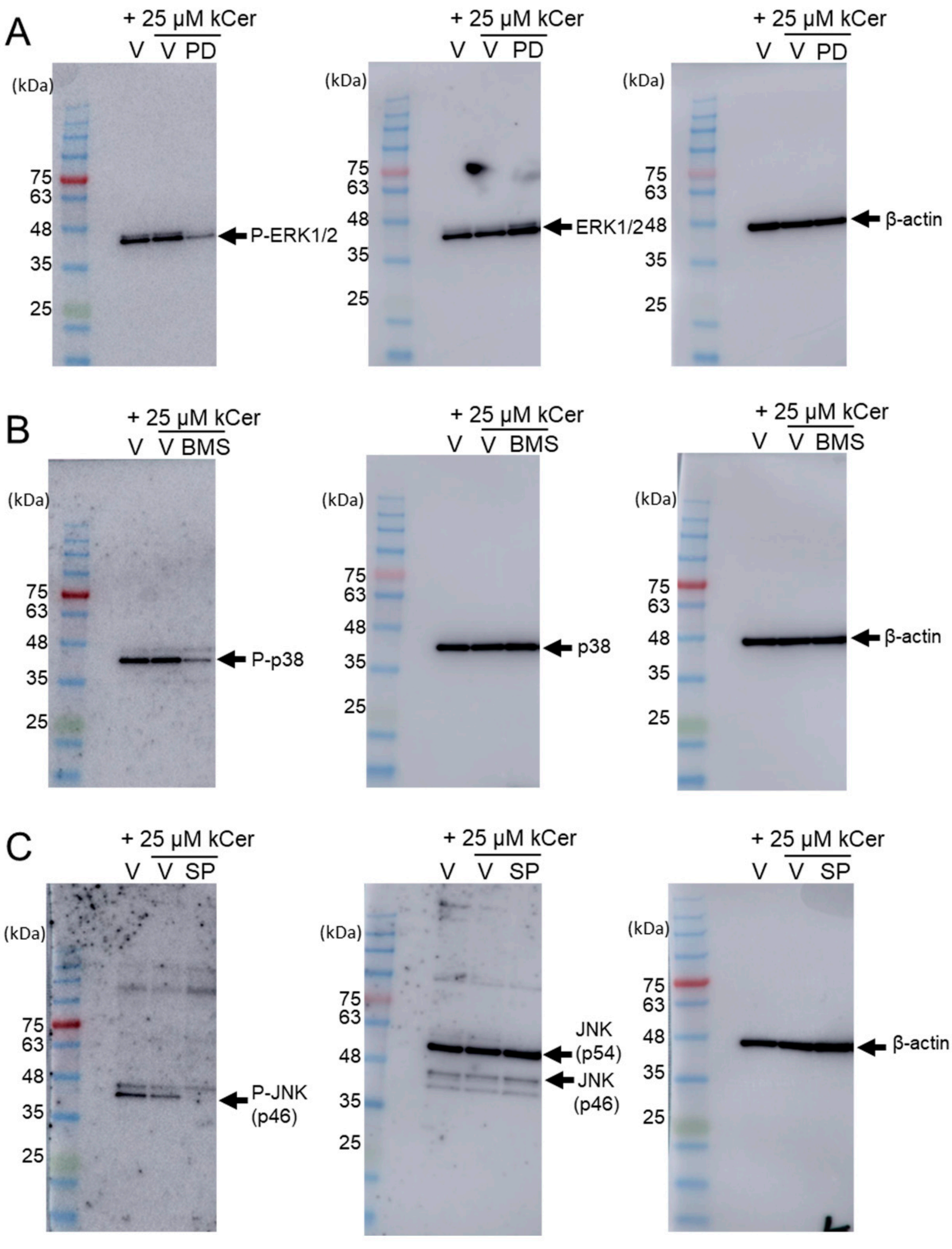

Figure S2. Western blot images used to generate Fig. 3. NHEKs were incubated with 25 μM kCer and each MAPK inhibitor: 25 μM PD98059 (**A**), 10 μM BMS-582949 (**B**), or 25 μM SP600125 (**C**). After 1 h, cell lysates were prepared and subjected to western blot analysis. V: vehicle, PD: PD98059 (MEK1/2 inhibitor), BMS: BMS-582949 (p38 inhibitor), SP: SP600125 (JNK inhibitor).

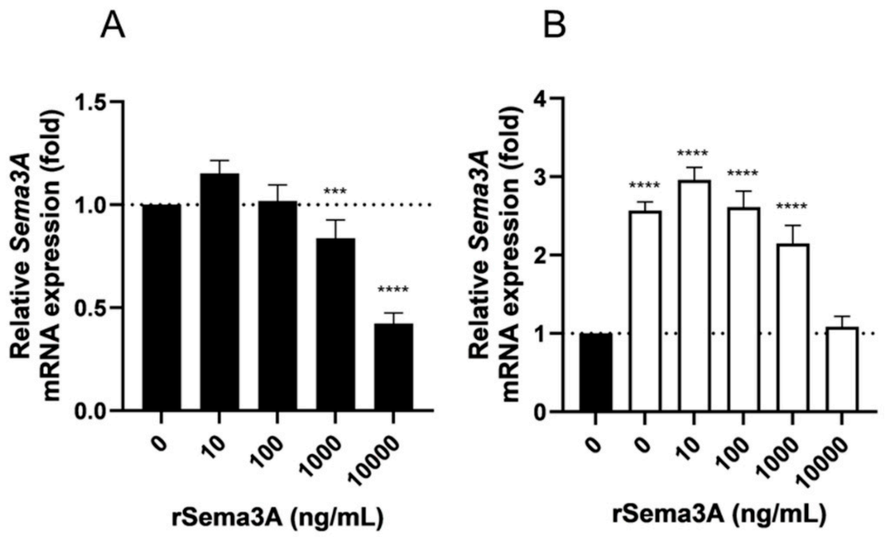

Figure S3. Recombinant Sema3A protein suppresses *Sema3A* mRNA expression in NHEKs. NHEKs were incubated with recombinant Sema3A (rSema3A; 10–10,000 ng/mL) in the presence of 0.1 mM (A) or 1.4 mM calcium (B) at 37°C for 12 h. *Sema3A* mRNA expression was examined by quantitative real-time PCR and normalized to RPS18. (**A**) High concentrations of rSema3A (1000 and 10,000 ng/mL) significantly suppressed *Sema3A* mRNA expression. (**B**) *Sema3A* mRNA expression was increased by 1.4 mM calcium, whereas a high concentration of rSema3A (10,000 ng/mL) suppressed this increase. All results are expressed as the mean ± S.D. of three independent experiments (vs. rSema3A 0 ng/mL, 0.1 mM calcium; one-way ANOVA followed by Dunnett's test).

Table S1. Human real-time PCR primers.

Primer name	Sequence
hRPS18	(forward) 5'-TTTGCGAGTACTCAACACCAACATC-3'<br>(reverse) 5'-GAGCATATCTTCGGCCCACAC-3'
hSema3A	(forward) 5'-ACCCAACTATCAATGGGTGCCTTA-3'<br>(reverse) 5'-AACACTGGATTGTACATGGCTGGA-3'
hc-Jun	(forward) 5'-CCCCAAGATCCTGAAACAGA-3'<br>(reverse) 5'-CCGTTGCTGGACTGGATTAT-3'
hc-Fos	(forward) 5'-AGAATCCGAAGGGAAAGGAA-3'<br>(reverse) 5'-CTTCTCCTTCAGCAGGTTGG-3'
hROR	(forward) 5'-AAATCGCATCTGGAAACCTG-3′<br>(reverse) 5'-TTGGCAAACTCCACCACATA-3′

Table S2. Murine real-time PCR primers.

Primer name	Sequence
mRPS18	(forward) 5'- CCAAGAAGGGAAGACGACTG -3'<br>(reverse) 5'- CGGATGAACTGACTGAGCAA -3'
mSema3A	(forward) 5'- ATGGCATTGACACCCATTTT -3'<br>(reverse) 5'- GGGACCATCTCTGTGAGCAT -3'
